# Bronchoalveolar lavage fluid and lung biopsy tissue metagenomic next-generation sequencing in the diagnosis of pulmonary cryptococcosis

**DOI:** 10.3389/fcimb.2024.1446814

**Published:** 2024-10-29

**Authors:** Jinbao Huang, Heng Weng, Ling Ye, Meiqin Jiang, Lulu Chen, Yangyu Li, Hongyan Li

**Affiliations:** ^1^ Department of Respiratory Medicine, The Affiliated People’s Hospital of Fujian University of Traditional Chinese Medicine, Fuzhou, China; ^2^ Department of Clinical Laboratory Medicine, The Affiliated People’s Hospital of Fujian University of Traditional Chinese Medicine, Fuzhou, China; ^3^ Department of Critical Care Medicine, The Affiliated People’s Hospital of Fujian University of Traditional Chinese Medicine, Fuzhou, China

**Keywords:** pulmonary cryptococcosis, metagenomic next-generation sequencing, *Cryptococcus neoformans*, lung biopsy tissue, bronchoalveolar lavage fluid

## Abstract

**Objective:**

To evaluate the diagnostic value of metagenomic next-generation sequencing (mNGS) in pulmonary cryptococcosis (PC) using bronchoalveolar lavage fluid (BALF) and lung biopsy tissue specimens.

**Methods:**

In this retrospective study, 321 patients diagnosed with lower respiratory tract diseases who underwent mNGS using BALF and LBT samples, between January 2021 and December 2023 were included. Individuals were classified into PC and non-PC groups according to the diagnostic criteria for PC, and conventional fungal cultures were performed. A serum/BALF cryptococcal antigen (CrAg) test was performed in some patients with PC. The diagnostic efficiencies of three methods for PC (mNGS, conventional culture, and CrAg) were compared. Additionally, two mNGS methods were used in this study: original mNGS (OmNGS, testing time from January 2021 to December 2022) and modified mNGS (MmNGS, testing time from January to December 2023). The diagnostic efficiency of the two mNGS methods on PC was simultaneously compared.

**Results:**

Among the 321 patients, 23 (7.2%) had PC and 298 (92.8%) did not. Compared with the composite reference standard for PC diagnosis, the sensitivity, specificity, and accuracy of mNGS for PC were 78.3% (95% confidence interval [CI], 55.8%–91.7%), 98.7% (95% CI, 96.4%–99.6%), and 97.2% (95% CI, 94.7%–98.7%), respectively. The sensitivity of mNGS was similar to that of CrAg (80.0%, 12/15) (*P* > 0.05). The diagnostic sensitivity of both mNGS and CrAg was higher than that of conventional culture (35.0%, 7/20) (*P* = 0.006, *P* = 0.016), and the combined detection of mNGS and CrAg further improved the diagnostic sensitivity of PC (93.3%, 14/15). The area under the receiver operating characteristic curve of mNGS was superior to that of conventional culture (0.885 vs. 0.675). In addition, the diagnostic sensitivity of PC was higher than that of OmNGS (*P* = 0.046).

**Conclusion:**

The sensitivity of mNGS is better than that of conventional culture. The combination of mNGS and CrAg improves the testing sensitivity of *Cryptococcus*. MmNGS could further improve the detection of *Cryptococcus*. Conventional PC detection methods are indispensable and mNGS can be used as a rapid and accurate auxiliary diagnostic method for PC.

## Introduction

Pulmonary cryptococcosis (PC) is an invasive fungal infection caused by *Cryptococcus* spp. The main fungal species were *Cryptococcus neoformans* (*C. neoformans*) and *Cryptococcus gattii*. Lung disease in humans is caused by the inhalation of *Cryptococcus* spores or yeasts (propagans) from the external environment (Botts and Hull, 2010; May et al., 2016; Montoya et al., 2021). Studies have shown that PC is mainly found in immunosuppressed hosts such as those with HIV/AIDS, cancer, rheumatic immune diseases, diabetes, and basic pulmonary diseases (Mirza et al., 2003; Kiertiburanakul et al., 2006). However, in recent years, the number of patients with PC with seemingly normal immune function has gradually increased (Lan et al., 2016; Liu et al., 2016; Chen et al., 2021), ranking third among pulmonary fungal diseases (Liu et al., 2011). Because of the complex and diverse clinical symptoms and imaging manifestations of PC, it is easily confused with pneumonia, tuberculosis, lung tumors, and other diseases, making early diagnosis difficult (Setianingrum et al., 2019).

Conventional detection methods for PC include smear staining and fungal cultures of respiratory tract specimens. Due to the influence of culture conditions and technical restrictions, there are shortcomings, such as a low positive rate and prolonged time of reporting, which cannot meet the needs of clinical diagnosis (Shirley and Baddley, 2009). In the past, the diagnosis of PC mainly depended on the pathological examination of lung tissue biopsy; however, limitations such as a long pathological diagnosis cycle and high technical requirements for pathological diagnosis personnel were observed, and it was not suitable for all patients because of its invasive nature (Zheng et al., 2021; Wang et al., 2022b). In recent years, the detection methods of cryptococcal antigen (CrAg) in serum have greatly improved the diagnostic efficiency of PC, especially the lateral flow immunoassay, which is convenient and easy to perform and has a high specificity (97.7–100%). The diagnostic sensitivity can reach 68.8–100% (McMullan et al., 2012; Chen et al., 2021; Yan et al., 2021; Shi et al., 2023), which has been widely promoted and applied in domestic clinical practice.

However, although the serum CrAg test has played an important diagnostic role, approximately 23.4–31.2% of patients still have false negatives (Yan et al., 2021; Hu et al., 2022; Shi et al., 2023), and there are certain limitations in the diagnosis of PC. Metagenomic next-generation sequencing (mNGS), a new experimental technology, has shown remarkable diagnostic ability for respiratory infections owing to its powerful pathogen detection ability (Zheng et al., 2021; Shi et al., 2022a; Wu et al., 2024). A meta-analysis of 1248 bronchoalveolar lavage fluid (BALF) specimens showed that the comprehensive diagnostic sensitivity and specificity of mNGS for lower respiratory tract infections (LRTIs) reached 89% and 90%, respectively, which is advantageous over conventional microbial diagnostic techniques (Guo et al., 2023). Another prospective study of 133 surgical lung specimens showed that mNGS had a sensitivity and specificity of 77.6% and 97.6%, respectively, for the diagnosis of LRTIs and could detect more fungi than the composite reference standard (Guo et al., 2021). Wang et al. (Wang et al., 2020) found that the diagnostic positivity rate of mNGS in 21 cases of mycotic pneumonia was significantly higher than that of conventional culture (90.5% vs. 4.8%). Compared to conventional microbiological tests, mNGS has a higher diagnostic performance in diagnosing pulmonary invasive fungal diseases (Wang et al., 2022a; Huang et al., 2024). In terms of cryptococcal infection, several clinical case reports have evaluated the application of mNGS in the diagnosis of cryptococcal meningitis (CM) (Xing et al., 2020; Jin et al., 2022; Shi et al., 2022c; Zhang et al., 2023), which can be an effective tool for CM diagnosis using cerebrospinal fluid (CSF) (Zhang et al., 2023). Preliminary data by Ramachandran et al. (Ramachandran et al., 2019) also suggested that mNGS has potential utility in detecting *Cryptococcus* in the central nervous system, even at a very low abundance. Simultaneously, occasional case reports and small case series have explored the ability of mNGS to detect *Cryptococcus* in human biopsy tissues (Guo et al., 2021; Wang et al., 2022b, 2022c; Liu et al., 2024a) and BALF (Zhang et al., 2024), suggesting that the histopathological combination with mNGS helps improve the diagnosis rate of cryptococcal infections and guide clinical treatment (Liu et al., 2024a). Moreover, BALF mNGS technology can be used to identify *Cryptococcus* in a non-invasive manner, providing a new rapid diagnostic method for PC (Su et al., 2022).

As an unbiased, fast, and highly sensitive microbial diagnostic method, mNGS is a popular diagnostic tool for infectious diseases, including respiratory and other systemic infections (Li et al., 2021; Kan et al., 2024). Nevertheless, with the exception of a few case reports, clinical studies evaluating the diagnostic ability of mNGS for PC are limited. Therefore, we reviewed the clinical data of 321 patients with lower respiratory diseases (LRTDs) who underwent mNGS to analyze and evaluate the potential value of this technique in the diagnosis of PC.

## Materials and methods

### Patients

The data of patients with initially suspected LRTIs who underwent mNGS detection and were finally diagnosed with LRTDs between January 2020 and December 2023 in our hospital were retrospectively analyzed. Patients meeting the following criteria were included in the study: (1) age ≥ge years; (2) patients with final diagnosis with LRTDs; (3) patients with BALF and lung biopsy tissue (LBT) samples receiving mNGS DNA testing; (4) all patients or entrusted agents agreed to undergo mNGS testing and provide signed informed consent. The exclusion criteria were as follows: (1) patients with pulmonary lesions whose final diagnosis was unclear; (2) patients with missing critical medical data; (3) patients with samples other than BALF and LBT; and (4) patients with unqualified quality control of mNGS test samples.

All patients tested for mNGS were screened, and the final diagnosis of LRTDs was confirmed based on the diagnostic criteria for LRTIs and non-LRTIs (Huang et al., 2023). All patients with LRTDs who fully met the inclusion criteria were then divided into PC patients and non-PC patients according to the diagnostic criteria for PC as follows (Huston and Mody, 2009; Shirley and Baddley, 2009; Donnelly et al., 2020; Su et al., 2022; Chang et al., 2024): (1) Definite diagnosis: the patient had typical clinical features and imaging findings of PC, and *Cryptococcus* was detected by pathological examination of lung tissue biopsy or smear/culture of lung puncture specimens (tissue, lung puncture fluid). (2) Clinical diagnosis: The patient had typical clinical features and imaging findings of PC, and the anti-cryptococcal therapy proved effective, and at least one of the following conditions was met: 1) serum/BALF CrAg test was positive; 2) sputum, BALF, blood or CSF smear/culture detected *Cryptococcus*; 3) BALF, or lung tissue samples were positive for *Cryptococcus* by mNGS. This study was conducted in accordance with the Declaration of Helsinki and approved by the Ethics Committee of the Affiliated People’s Hospital of Fujian University of Chinese Medicine. As this was a retrospective study, the requirement for informed consent was waived, and all data were analyzed anonymously.

### Conventional microbiological screening

In addition to the mNGS, the attending physician selected the appropriate conventional microbiological test content according to the patient’s clinical needs. These include bacterial, fungal smears and cultures, acetobacter smears, humoral cell Hexamidosilver staining of *Pneumocystis jirovecii*, polymerase chain reaction (PCR) of various pathogens, GeneXpert, BALF Galactomannan antigen (GM) and CrAg detection, serum 1, 3-β-D-glucan, GM, CrAg detection and various serum pathogen antibody detection. Test specimens included sputum, BALF, lung tissue, blood, and CSF.

### mNGS detection

BALF and LBT samples from all patients were sent to a genetic testing company (Hangzhou Jieyi Biological Company, Hangzhou, China) for mNGS DNA testing to identify pathogenic microorganisms. The mNGS testing procedures included specimen collection and pre-treatment, NGS masterTM (Cat.MAR002, MatriDx. Hangzhou, China) for automatic nucleic acid extraction, library construction, computer sequencing, database comparison, report interpretation, and prognosis tracking (Guo et al., 2021; Li et al., 2021; Kan et al., 2024). Using the Illumina sequencing platform and PCR-free library building technology, mNGS performs high-throughput sequencing of nucleic acids extracted from biological samples to obtain microbial species and abundance information contained in the samples. The detection procedure was as follows. First, 1.2 mL samples were homogenized and centrifuged, and DNA was extracted using a total nucleic acid extraction kit (Cat.MD013, MatriDx Biotech Corp. Hangzhou, China). A total DNA library preparation kit (Cat.MD001T, MatriDx Biotech Corp. Hangzhou, China) was used to prepare a sequencing library. Libraries were sequenced using a 75-cycle sequencing kit and an Illumina NextSeq 550 sequencer (San Diego, California, USA). Notably, 10–20 million reads were obtained for each sample, and bioinformatics analysis was performed using self-built analysis software (mngslibs), with the NCBI nt database and GenBank used as reference microbial databases (Benson et al., 2013). Detailed methods have been previously described (Huang et al., 2023). In this study, clinical specimens selected from 2021 to 2022 were detected using the above mNGS method called original mNGS (OmNGS).

In January 2023, the mNGS technology adopted in this study was innovated based on OmNGS and was named modified mNGS (MmNGS). The upgraded details are as follows: First, the self-developed sample shock crusher was improved. In addition to continuing to use the homogeneous wall-breaking method, the number and diameter of homogeneous medium glass beads were further adjusted to the optimal proportion to meet the needs of microorganisms with different diameters and different cell wall thicknesses, improve the physical wall-breaking ability, and strengthen the wall-breaking ability of refractory pathogens to increase the detection sensitivity. Second, in contrast to the selective nucleic acid removal technology adopted by OmNGS in our previous study (Huang et al., 2023), MmNGS adopts forward broad-spectrum enrichment technology (pathogen enrichment) (Shi et al., 2022b) and uses a self-developed super-multiple alignment algorithm to analyze the sequence conservation of tens of thousands of microorganisms in a self-built microbial database. The algorithm can select a relatively conserved region suitable for broad-spectrum enrichment and design a primer group that can achieve broad-spectrum enrichment through a specific multi-primer design. This primer set is not specific to a specific species and can enrich the nucleic acids of multiple microbial species simultaneously, while the enriched sequences still undergo species-specific analyses. This technique eliminates the restriction of microorganisms and host content on sensitivity, such that the pathogen detection efficiency of low-concentration samples is as free as possible from the interference of host content.

### Pathogenicity interpretation of detected microorganisms in the mNGS report

The results of the mNGS report were independently interpreted by three senior professionals with bioinformatics knowledge and engaged in pulmonary medicine or clinical microbiology, combined with the medical history of patients, imaging data, pathological findings, other laboratory test results, and clinical treatment response (Li et al., 2021). In cases of disagreement regarding the pathogenicity of the detected *Cryptococcus*, a consensus was reached through discussion and consultation. The composite reference standard for diagnostic efficiency comparison with different detection methods for *Cryptococcus* included the results from all laboratory detections (including CrAg and mNGS), imaging manifestations, pathological examinations, and clinical comprehensively analysis and judgment (Guo et al., 2021; Su et al., 2022).

### Statistical analysis

Statistical software (SPSS 19.0, IBM SPSS Inc., Chicago, USA) was used for the data analysis. Measurement data are expressed as median (interquartile distance), and comparisons between the OmNGS and MmNGS groups for sequence number and relative abundance of *Cryptococcus* at the species level were performed using non-parametric rank sum tests. Count data are expressed as percentages (%), and the diagnostic performance of the different detection methods is expressed in terms of sensitivity, specificity, and accuracy. The diagnostic efficacy of mNGS, CrAg detection, and traditional culture methods, and OmNGS, MmNGS, and CrAg detection methods were compared using the chi-square test or Fisher’s exact probability methods. The area under the curve (AUC), which was used to evaluate and compare the performances of mNGS and conventional fungal culture, was determined using receiver operating characteristic (ROC) curve analysis. Statistical significance was set at *P* < 0.05.

## Results

### Characteristics of enrolled patients

**Figure 1:**
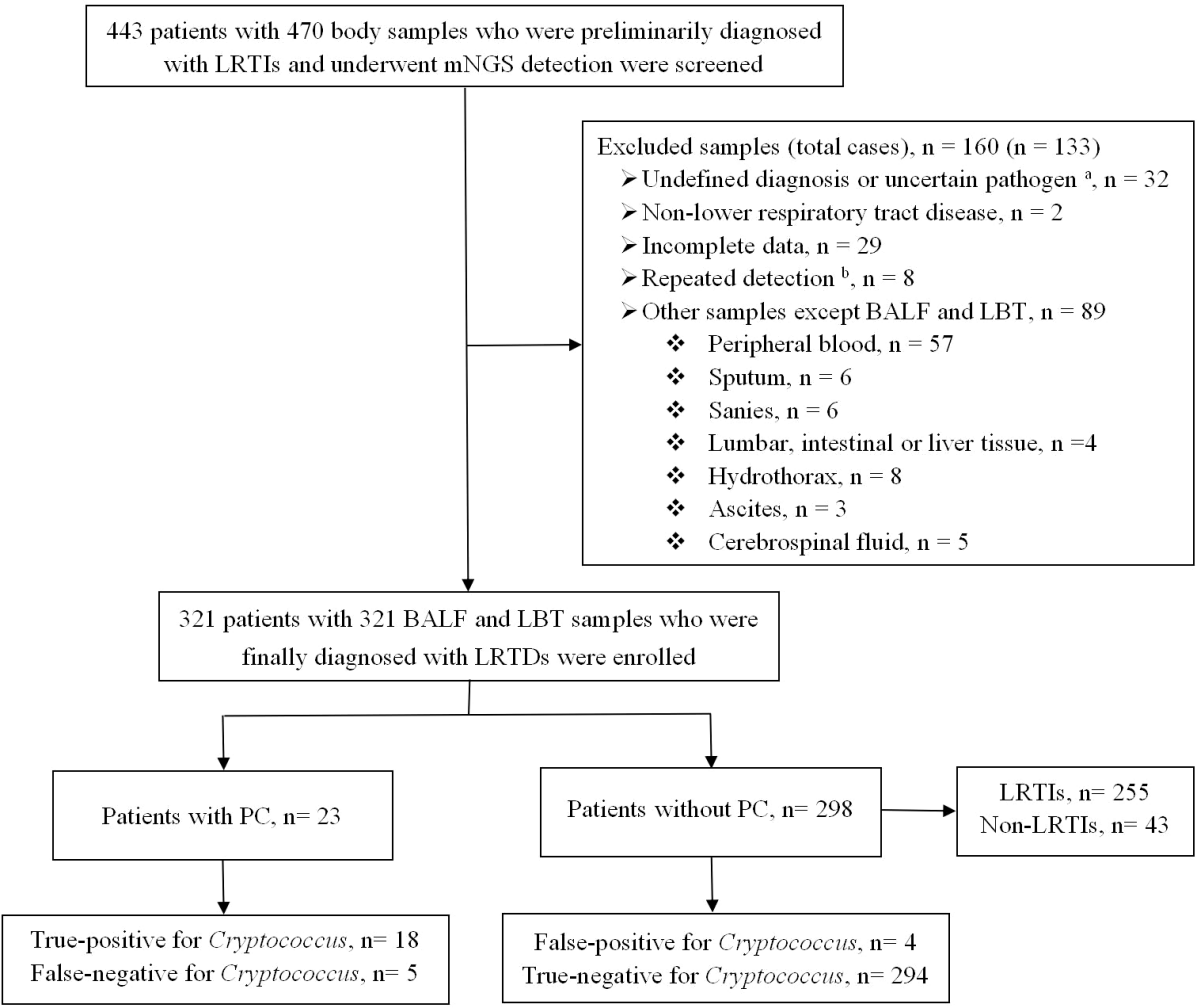
Flowchart of the enrolled patients. BALF, bronchoalveolar lavage fluid; LBT, lung biopsy tissue; LRTIs, lower respiratory tract infections; LRTDs, lower respiratory tract diseases; mNGS, metagenomic next-generation sequencing; PC, pulmonary cryptococcosis. ^a^ Uncertain pathogen, unclear whether the detected pathogen is colonizing or pathogenic bacteria. ^b^ The same patient was tested multiple times, and the result of the first lower respiratory tract specimen was selected.

Between January 2020 and December 2023, 470 human specimens from 443 patients were tested using mNGS. Finally, 321 lower respiratory tract specimens from 321 patients with LRTDs including 278 LRTIs and 43 non-LRTIs met the inclusion criteria and were further divided into 23 (6.9%) patients with PC and 298 (92.8%) patients without PC, according to the diagnostic criteria for PC (**Figure 1**).

Among the 321 samples tested using mNGS, 294 (91.6%) were BALF and 27 (8.4%) were LBT samples. mNGS samples from the 23 PC cases included 11 BALF (47.8%) and 12 LBT (52.2%) samples.

As shown in **Table 1**, of the 23 patients with PC, 19 (82.6%) had underlying diseases, 15 (65.2%) had immunosuppressive diseases and/or underlying lung diseases, and 2 (8.7%) had a history of high-risk environmental exposure (breeding birds). The median age of the patients was 59 (48, 68) years, and there were 14 (60.9%) males. Clinical manifestations included cough (11 cases), sputum (seven cases), fever (three cases), chest tightness (one case), shortness of breath (two cases), fatigue (one case), headache (one case). The remaining 10 cases (43.5%) were asymptomatic. Chest computed tomography (CT) showed that 20 cases (87.0%) had single or multiple pulmonary nodules and mass shadows, and 3 cases (13.0%) had consolidation shadows. Lung biopsies were performed in 13 cases, of which 12 cases (92.3%) of percutaneous lung biopsy (PNLB) were pathologically confirmed to have cryptococcal infection, and one case of transbronchial lung biopsy was pathologically negative. Of the overall cohort, 254 (79.1%) patients had underlying diseases including 146 immunocompromised diseases. The median age of the patients was 63 (54, 71) years and 169 (66.6%) cases were males. There were 67 patients (20.9%) without underlying diseases, among who 43 (64.2%) were males. The median age of the patients was 57 (41, 66) years.

**Table 1:** Clinical data of 23 cases of PC with pathogen detected using mNGS.

Case	Age/sex	Underlying diseases/Environmental exposure	Imaging findings	Diagnosic type	Prognosis
[1] [2] [3] [4] [5] [6] [7] [8] [9] [10] [11] [12] [13] [14] [15] [16] [17] [18] [19] [20] [21] [22] [23]	39/M 35/M 58/F 58/F 33/F 46/M 79/F 59/M 58/M 48/M 68/F 59/M 78/M 54/M 49/F 70/M 71/M 64/F 55/F 75/M 63/M 27/M 65/F	None Breeding parrot Breeding mynah Diabetes mellitus AIDS Hypertension Malignant lymphoma Pneumoconiosis, breast cancer after surgery and thymus tumor after surgery Bronchiectasis, hypertension None Chronic atrophic gastritis Diabetes mellitus, hypertension Nasopharyngeal cancer after chemotherapy and OP with hormonotherapy Chronic atrophic gastritis and DU Breast cancer after surgery and chemotherapy COPD with glucocorticoid therapy and gastric cancer Chronic renal insufficiency and bladder tumor after surgery Diabetes mellitus Cervical cancer after chemoradiotherapy Hypertension COPD, pneumoconiosis, and hypertension Enterophthisis ANCA-associated pulmonary vasculitis with glucocorticoid therapy, hepatitis B carriers, and bronchiectasis	Multiple nodules in bilateral lung Multiple patches and nodules in LLL Multiple nodules with cavities in bilateral lung Multiple nodules in bilateral lung Patchy consolidation in RUL Multiple nodules in bilateral lung Multiple nodules in bilateral lung Multiple patches and nodules in bilateral lung Multiple patches and nodules with right pleural effusion Multiple nodules and fibrotic linear opacities in bilateral lung Solitary nodule in RLL Multiple patches and nodules in RL Multiple nodules with cavities in LL Multiple nodules in bilateral lung Solitary mass shaw in RLL Solitary nodule in RLL Multiple patches and nodules in bilateral lung Flake consolidation in RLL Multiple nodules and mass in RLL Multiple nodules in bilateral lwer lung Multiple nodules in bilateral lwer lung Solidary mass shadow in LLL Multiple patches and consolidations in bilateral lwer lung	Definite diagnosis Clinical diagnosis Definite diagnosis Clinical diagnosis Clinical diagnosis Clinical diagnosis Clinical diagnosis Definite diagnosis Definite diagnosis Definite diagnosis Definite diagnosis Definite diagnosis Clinical diagnosis Clinical diagnosis Definite diagnosis Definite diagnosis Clinical diagnosis Clinical diagnosis Definite diagnosis Definite diagnosis Clinical diagnosis Definite diagnosis Clinical diagnosis	Cure Cure Cure Cure Cure Cure Cure Cure Cure Cure Cure Cure Cure Cure Cure Cure Cure Cure Cure Cure Cure Cure Death *

AIDS, acquired immune deficiency syndrome; ANCA, anti-neutrophil cytoplasmic antibodies; CM, Cryptococcal meningitis; COPD, chronic obstructive pulmonary disease; DU, duodenal ulcer; LL, left lung; LLL, left lower lung; mNGS, metagenomic next-generation sequencing; OP, organizing pneumonia; PC, pulmonary cryptococcosis; RL, right lung; RLL, right lower lung; RUL, right upper lung.

* The patient died because of the progression of refractory hypoxemia and multiple organ failure secondary to the underlying anti-neutrophil cytoplasmic antibody-associated vasculitis.

According to the diagnostic criteria for PC, 12 cases (52.2%) were definite and 11 cases (47.8%) met the clinical diagnosis. The latter all had typical clinical manifestations (**Table 1**) and met the following conditions (**Tables 2**, **3**): (1) serum CrAg detection was positive in Case 2; (2) serum/BALF CrAg detection was positive in Cases 4, 6, 7, 13, 18, and 23 and BALF mNGS were also positive for *Cryptococcus*; (3) blood and CSF cultures and BALF mNGS test were positive for *Cryptococcus* in Cases 5; (4) serum CrAg detection was positive in Cases 14 and 21 and *Cryptococcus* was also detected by culture of sputum/BALF specimens and BALF mNGS; and (5)*Cryptococcus* was detected by BALF mNGS in Case 17.

**Table 2:** Testing results of mNGS of 23 patients with PC.

Case number	mNGS method	*Cryptococcus* species	*Cryptococcus* species reads	*Cryptococcus* species relative abundance (%)*	Pulmonary co-infectious pathogen
[1] [2] [3] [4] [5] [6] [7] [8] [9] [10] [11] [12] [13] [14] [15] [16] [17] [18] [19] [20] [21] [22] [23]	OmNGS OmNGS OmNGS OmNGS OmNGS OmNGS OmNGS OmNGS OmNGS OmNGS OmNGS OmNGS OmNGS MmNGS MmNGS MmNGS MmNGS MmNGS MmNGS MmNGS MmNGS MmNGS MmNGS	*C. neoformans* var. *grubii* Negative Negative *C. neoformans* var. *grubii* *C. neoformans* var. *grubii* *C. neoformans* *C. neoformans* var. *grubii* *C. neoformans* var. *grubii* Negative *C. neoformans* var. *grubii* Negative Negative *C. neoformans* var. *grubii* *C. neoformans* species complex *C. neoformans* *C. neoformans* var. *grubii* *C. neoformans* species complex *C. neoformans* var. *grubii* *C. neoformans* var. *grubii* *C. neoformans* *C. neoformans* var. *grubii* *C. neoformans* species complex *C. neoformans*	1261 NA NA 24 2 2 1408 46 NA 24 NA NA 1 13 216 254 270 65 204 31 3 1863 1795	48.3 NA NA < 0.01 0.29 0.07 19.4 17.2 NA 11.1 NA NA 0.14 < 0.01 0.15 0.21 0.17 0.06 0.18 0.12 0.53 8.57 0.35	None None None None *Talaromyces marneffei* None None None None None None None None None None *Pseudomonas aeruginosa* None None None None MTB None *Aspergillus aflatus*/*Aspergillus oryzae* and *Pneumocystis jirovecii*

*Cryptococcus neoformans*, *C. neoformans*; MTB, *Mycobacterium tuberculosis complex*; MmNGS, modified mNGS; mNGS, metagenomic next-generation sequencing; NA, not available; OmNGS, original mNGS; PC, pulmonary cryptococcosis; PJ, *Pneumocystis jirovecii*.

*Relative abundance was defined as number of *Cryptococcus* species reads divided by total reads in a sample.

**Table 3:** Comparison of the results of different *Cryptococcus* detection methods.

Case	mNGS/Specimen	CrAg detection/Specimen	Culture/Specimen	Pathology/Specimen
[1] [2] [3] [4] [5] [6] [7] [8] [9] [10] [11] [12] [13] [14] [15] [16] [17] [18] [19] [20] [21] [22] [23]	Positive/PNLB Negative/BALF Negative/PNLB Positive/BALF Positive/BALF Positive/BALF Positive/BALF Positive/PNLB Negative/PNLB Positive/PNLB Negative/PNLB Negative/PNLB Positive/BALF Positive/BALF Positive/PNLB Positive/PNLB Positive/BALF Positive/BALF Positive/PNLB Positivie/PNLB Positive/BALF Positive/PNLB Positive/BALF	Positive/Serum Positive/Serum Negative/Serum Positive/Serum NA Positive/BALF Positive/Serum and BALF NA NA Positive/Serum NA NA Positive/Serum and BALF Positive/Serum NA NA NA Positive/Serum Positive/Serum Negative/Serum Positive/Serum Negative/Serum Positive/Serum	NA Negative/BALF Negative/BALF Negative/BALF Positive/Peripheral blood, CSF Negative/BALF Negative/BALF Positive/Lung puncture fluid NA Positive/CSF Negative/Lung puncture fluid NA Negative/BALF Positive/BALF Negative/Sputum Positive/Lung puncture fluid Negative/BALF Negative/BALF Positive/Lung puncture fluid Negative/Lung puncture fluid Positive/Sputum, Negative/BALF Negative/Lung puncture fluid Negative/BALF	Positive/PNLB NA Positive/PNLB NA NA Negative/TBLB NA Positive/PNLB Positive/PNLB Positive/PNLB Positive/PNLB Positive/PNLB NA NA Positive/PNLB Positive/PNLB NA NA Positive/PNLB Positive/PNLB NA Positive/PNLB NA

BALF, bronchoalveolar lavage fluid; CSF, cerebrospinal fluid; mNGS, metagenomic next-generation sequencing; NA, not available; PNLB, percutaneous needle lung biopsy tissue; TBLB, transbronchial lung biopsy.

Of the 23 cases, 21 (86.4%) with cryptococcal lesions involving the lungs had isolated PC, four of which were co-infected with other pathogens. Two cases (9.1%) had disseminated cryptococcal infection, of which one case was PC combined with CM, and the other case was PC combined with CM, cryptococcal septicemia, and *Talaromyces marneffei*. After treatment, 22 patients were cured, and only one patient (Case 23 in **Table 1**) died due to the progression of refractory hypoxemia and multiple organ failure secondary to the underlying disease of anti-neutrophil cytoplasmic antibody-associated vasculitis.

### Results of mNGS detection

Among 255 LRTIs without proven or clinical PC, pathogens were identified using mNGS in 166 cases, as shown in **Supplementary Tables 1, 2**. Of the 23 PC cases, 18 (78.3%) tested positive for *Cryptococcus* by mNGS, as shown in **Tables 2**, **3**. The average sequence number and relative abundance of *Cryptococcus* at the species level were 55.50 (10.50, 517.75) and 0.20% (0.11%, 9.20%), respectively. In addition to *Cryptococcus*, *Talaromyces marneffei*, *Pseudomonas aeruginosa*, *Mycobacterium tuberculosis*, *Aspergillus aflatus*/*Aspergillus oryzae*, and *Pneumocystis jirovecii* were detected in the four BALF samples, indicating mixed infection. There was no significant difference in the sensitivity (90.9%, 10/11 vs. 66.7%, 8/12) of *Cryptococcus* detection by mNGS between the BALF and LBT samples (*P* = 0.317).

OmNGS was used to detect 13 cases of PC from 2021 to 2022, of which 8 (61.5%) were positive for *Cryptococcus*. The mean sequence number and relative abundance of *Cryptococcus* at the species level were 24.00 (2.00, 957.25) and 5.70% (0.09%, 18.85%). In 2023, 10 cases of PC were detected using MmNGS, and all cases (100%) were positive for *Cryptococcus*. The average sequence number and relative abundance of *Cryptococcus* at the species level were 210.00 (26.50, 651.25) and 0.18% (0.11%, 0.4%). The sequence numbers and relative abundances of *Cryptococcus* species detected using OmNGS and MmNGS were not significantly different (*P* = 0.146 and *P* = 0.274, respectively). As shown in **Table 4**, the sensitivity of MmNGS for PC diagnosis was higher than that of OmNGS (*P* = 0.046), whereas there were no significant differences in specificity or accuracy between MmNGS and OmNGS (*P* > 0.05). The sensitivity (83.3%, 5/6 vs. 42.9%, 3/7) of *Cryptococcus* by OmNGS in the BALF and LBT samples was not significantly different (*P* = 0.266).

**Table 4:** Comparison of different mNGS methods for *Cryptococcus* detection.

	OmNGS (n = 217)	MmNGS (n = 104)	*P*
Sensitivity (95% CI) Specificity (95% CI) Accuracy (95% CI)	61.5 (8/13) (32.3, 84.9) 98.5 (201/204) (95.4, 99.6) 96.3 (209/217) (92.9, 98.4)	100 (10/10) (62.5, 100) 98.9 (93/94) (93.4, 100) 99.0 (103/104) (94.8, 100)	0.046 1.000 0.133

CI, confidence interval; MmNGS, modified mNGS; mNGS, metagenomic next-generation sequencing; OmNGS, original mNGS.

Data are presented as n (%). Significance was determined by a Fisher’s exact probability test.

### Comparison of different detection methods for *Cryptococcus*

Among the 23 cases of PC, in addition to BALF or LBT mNGS detection, 20 patients underwent simultaneous conventional fungal culture of human specimens, of which 7 (35.0%) were identified *C. neoformans*; the positive specimens included sputum (one case), BALF (one case), lung puncture fluid (three cases), blood (one case), and CSF (two cases). Among them, one case (Case 5 in **Table 3**) was positive for both CSF and blood, and the positivity rate of the lower respiratory tract specimens (sputum, BALF, and lung puncture fluid) was only 27.8% (5/18). Serum/BALF CrAg tests were performed simultaneously in 15 cases, of which 12 (80.0%) were positive for the cryptococcal antigen.

There was a significant difference in the detection sensitivity of mNGS, conventional culture, and CrAg in the diagnosis of PC (*P* = 0.004). The sensitivities of mNGS and CrAg were higher than that of conventional culture (*P* = 0.006 and *P* = 0.016, respectively), whereas there was no significant difference between mNGS and CrAg (*P* > 0.05). The sensitivity of the combined detection of mNGS and CrAg in the diagnosis of PC increased to 93.3% (14/15), whereas there was no statistically significant difference compared to the detection of mNGS and CrAg alone (*P* = 0.528). There were no significant differences in the specificity or accuracy between mNGS and conventional culture for PC diagnosis (*P* > 0.05) (**Table 5**). In addition, there was no significant difference in the sensitivity of OmNGS and MmNGS in diagnosing PC compared with CrAg (*P* = 0.410 and *P* = 0.250, respectively).

**Table 5:** Comparison of mNGS and Conventional culture for *Cryptococcus* detection.

	mNGS (n = 321)	Conventional fungal culture * (n = 318)	*P*
Sensitivity (95% CI) Specificity (95% CI) Accuracy (95% CI)	78.3 (18/23) (55.8, 91.7) 98.7 (294/298) (96.4, 99.6) 97.2 (312/321) (94.7, 98.7)	35.0 (7/20) (16.3, 59.1) 100 (298/298) (98.4, 100) 95.9 (305/318) (93.1, 97.8)	0.006 0.124 0.395

CI, confidence interval; mNGS, metagenomic next-generation sequencing.

* Samples included sputum, bronchoalveolar lavage fluid, lung puncture fluid, peripheral blood, and cerebrospinal fluid.

Data are presented as n (%). Significance was determined by the chi-square test or Fisher’s exact probability methods.

### ROC curves of mNGS and conventional fungal culture for PC diagnosis

**Figure 2:**
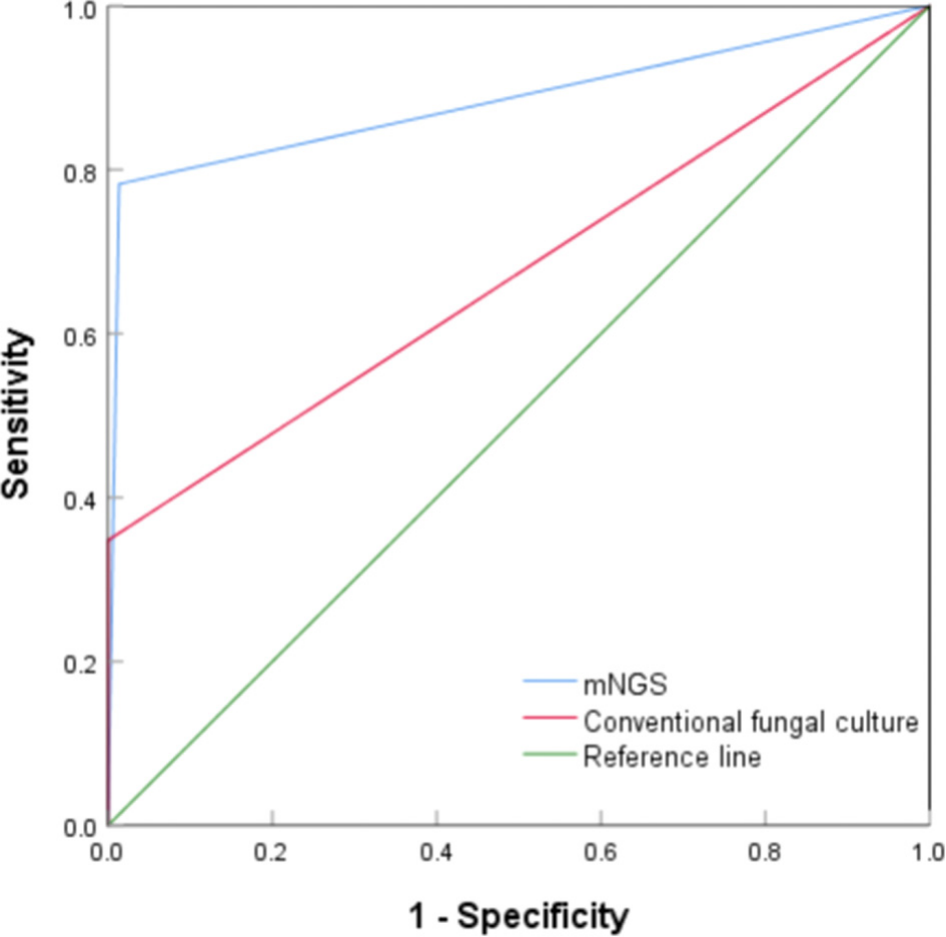
Receiver operating characteristic curve of metagenomic next-generation sequencing and conventional culture for diagnosis of pulmonary cryptococcosis.

The diagnostic value of mNGS and conventional fungal cultures for PC were compared using ROC curves. As shown in **Figure 2**, the areas under the curve (AUC) for mNGS and conventional fungal culture were 0.885 (95% CI, 0.784–0.985, *P* < 0.001) and 0.675 (95% CI, 0.527–0.823, *P* = 0.009), respectively. Compared with the composite reference standard for PC diagnosis, the sensitivity and specificity of mNGS and conventional fungal culture were 78.3% (95% CI, 55.8%–91.7%)/98.7% (95% CI, 96.4%–99.6%) and 35.0% (95% CI, 16.3%–59.1%)/100% (95% CI, 98.4%–100%), respectively.

### Analysis of four cases of LRTDs with false-positive for *Cryptococcus* detected using mNGS

*Cryptococcus* was detected using mNGS in the BALF of four patients with LRTDs. The sequence numbers and relative abundances of *Cryptococcus* at the species level were 1 (0.07%), 6 (0.02%), 4 (0.34%), and 7 (0.05%), respectively. The results were confirmed as false positives by comprehensive clinical judgment and were diagnosed as *Mycoplasma* pneumonia, tuberculosis, pulmonary metastasis of gastric cancer (Huang et al., 2023), and pulmonary embolism. Three patients (75%) had underlying immunosuppressive diseases, including nephrotic syndrome, diabetes mellitus, and advanced gastric adenocarcinoma, and one patient had atrial fibrillation. All patients were male and were 27, 55, 75, and 54 years old, except for one patient who died from advanced gastric cancer with lung metastasis, and the other three patients were cured with anti-*Mycoplasma*, anti-tuberculosis, and anticoagulant therapies.

### Example 1: true-positive for *Cryptococcus* in PC (Case 22 in **Table 2**)

**Figure 3:**
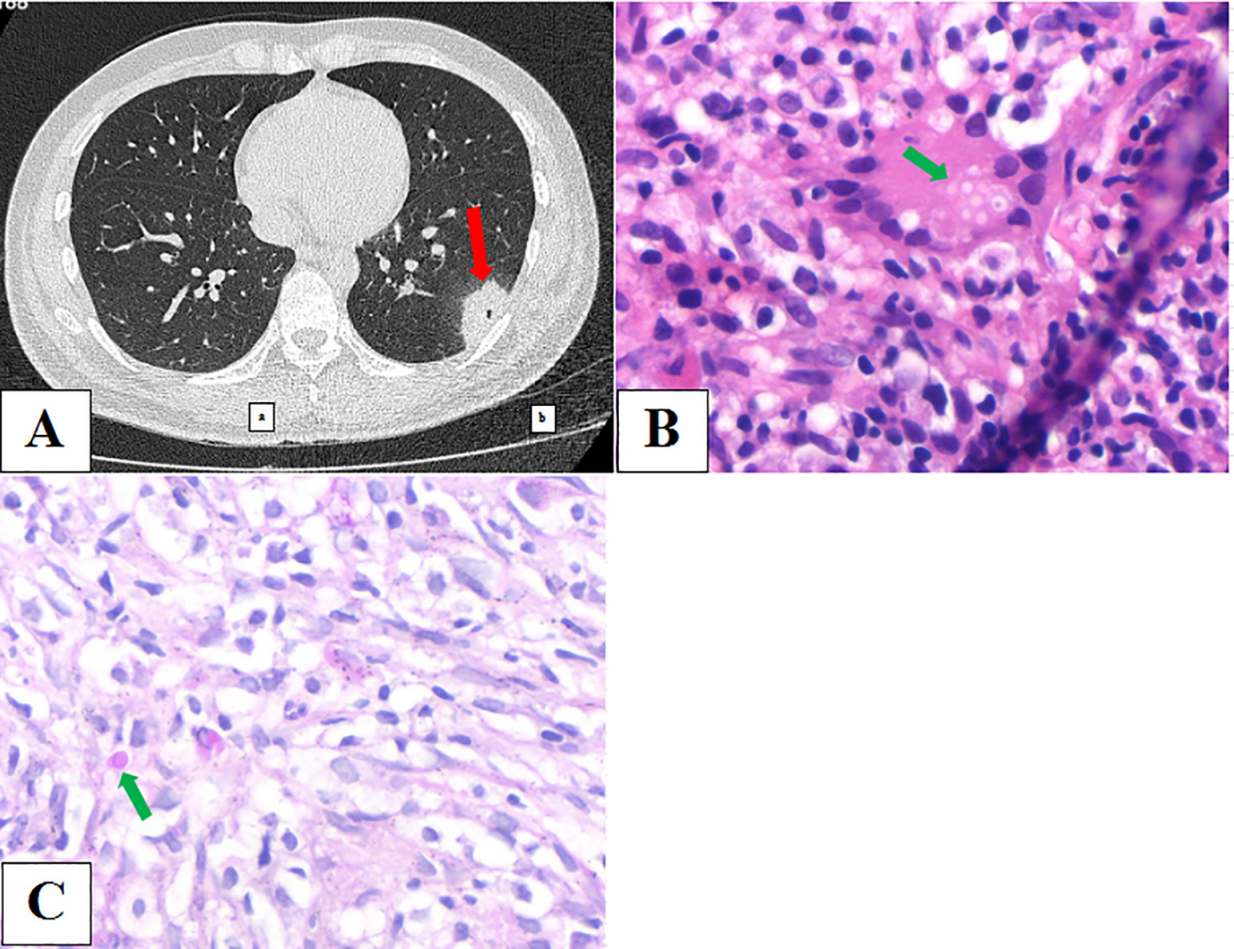
**(A)** Chest computed tomography image showing an isolated subpleural mass shadow of the left lower lung with solidary cavity and a surrounding halo sign. **(B, C)** Repeated pathological results of anatomic slices showed a small amount of *Cryptococcus* in individual megakaryocytes.

A 27-year-old male patient was admitted to our hospital on October 22, 2023, and underwent physical examination of a mass shadow in the lower left lung for 4 days. He underwent intestinal tuberculosis treatment 8 years previously. Routine blood examination revealed normal C-reactive protein (CRP) levels. The serum CrAg level was negative. Chest CT (**Figure 3A**) shows an isolated subpleural mass shadow in the left lower lung with solitary cavity and a surrounding halo sign. CT-guided pulmonary puncture biopsy was performed on the lower left pulmonary lesion, and lung pathology revealed a large amount of fibrin exudation in the alveolar cavity and diffuse interstitial fibrosis with large lymphocyte infiltration. The testing results of the LBT by mNGS (MmNGS) revealed *C. neoformans* complex (**Table 2**). After communicating with the Pathology Department, deep paraffin samples were obtained. Repeated pathological analysis of the anatomical slices showed a small amount of *Cryptococcus* in individual megakaryocytes (**Figures 3B, C**), consistent with the MmNGS findings. The patient was diagnosed with PC and was cured after regular antifungal treatment with fluconazole.

### Example 2: false-positive for *Cryptococcus* in *Mycoplasma pneumoniae*-related pneumonia

**Figure 4:**
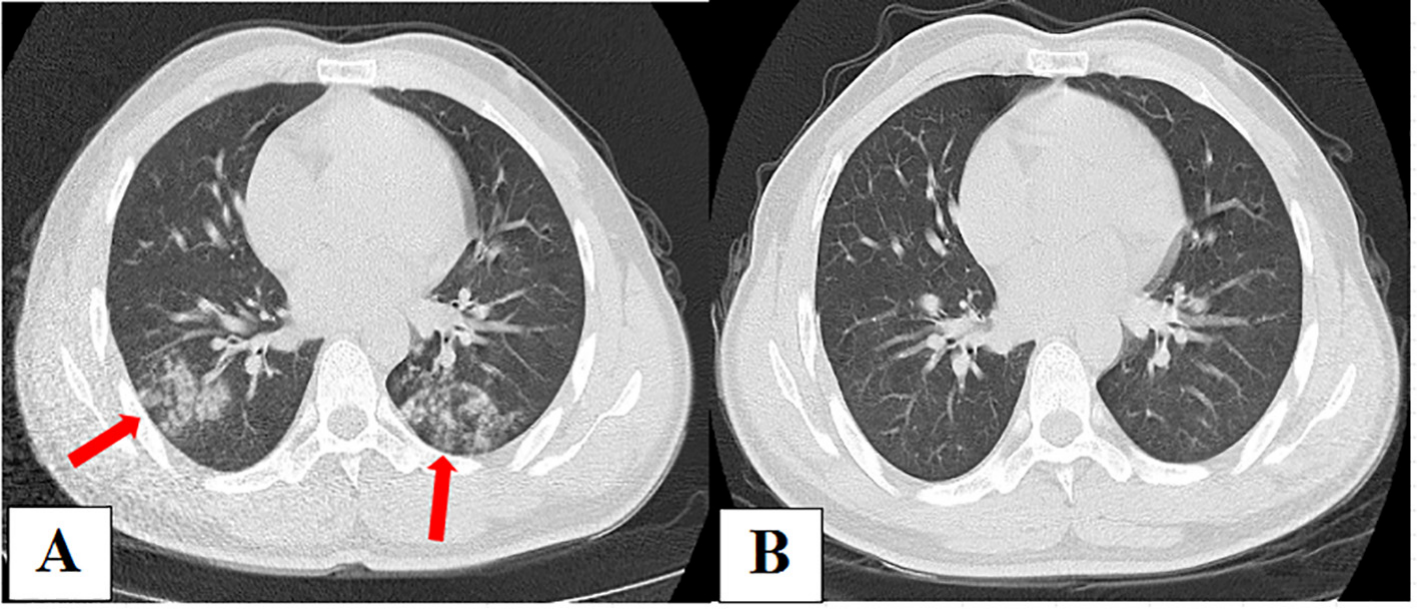
**(A)** Chest computed tomography (CT) on admission showing patchy and nodular shadows in both lungs. **(B)** After 3 weeks of antibiotic therapy, repeated chest CT showed almost complete absorption of lesions in both lungs.

A 30-year-old male was admitted to our hospital on January 7, 2021, with a cough, low fever, and edema of the face and lower extremities for 7 days. Nephrotic syndrome was confirmed via renal puncture after admission. CT scan on admission (**Figure 4A**) showed patchy and nodular shadows in both lungs. Results of routine blood examinations and T-lymphocyte subsets were normal. Serum CRP test was 17.91 mg/L. Serum GM and G tests were negative, and serum total *Mycoplasma* antibody was positive, with a titer of 1:40. Bronchoscopy was performed, and BALF GM and pathogen cultures were negative. The BALF mNGS (OmNGS) was positive for *C. neoformans*. The sequence number and relative abundance of *Cryptococcus* at the species level were 1 and 0.07%. *Mycoplasma pneumoniae*-related pneumonia with suspected PC was suspected. Moxifloxacin (400 mg, once daily) was initially administered to treat the infection, and the symptoms disappeared. After 3 weeks of antibiotic therapy, a repeat chest CT scan (**Figure 4B**) showed almost complete absorption of the lesions in both lungs. The diagnosis of *Mycoplasma* pneumonia was confirmed, and PC was ruled out. The result of *Cryptococcus* detection using mNGS in BALF samples was false positive, suggesting the presence of non-pathogenic fungi.

## Discussion

In this retrospective study, there were 23 cases of PC, of which 14 underwent lung biopsy and pathological examination, and 12 met the diagnostic criteria for PC, with a positivity rate of 92.3%, which is consistent with the literature (91.9%) (Chen et al., 2021). Serum/BALF CrAg was detected in 18 of 23 cases, with a positivity rate of 80.0%, similar to that reported in the literature (Hu et al., 2022; Shi et al., 2023). However, the sensitivity of conventional fungal cultures in various human specimens was only 35%, and the positivity rate of cultures in the lower respiratory tract specimens was lower (27.8%), which was similar to the study reported by Su et al (Su et al., 2022). Our results showed that approximately half of the patients with PC were confirmed via histopathology, and the remaining patients were diagnosed using mNGS, CrAg test, or culture combined with comprehensive clinical data. The sensitivity of mNGS for BALF and LBT samples was 78.3%. The result was similar to those reported by Wang et al. (Wang et al., 2020) (BALF specimens, 75.0%, 3/4) and Guo et al. (2021) (LBT specimens, 80.0%, 4/5), and similar to CrAg detection; however, the sensitivities of mNGS and CrAg detection were both higher than that of conventional culture (*P* < 0.05). Moreover, as shown in Cases 2, 20, and 22 (**Table 3**), the sensitivity of the combined detection of mNGS and CrAg further increased to 93.3%, indicating that the two methods are highly complementary. Additionally, the specificity of mNGS was as high as 98.7%, similar to that of Illumina platform sequencing by Guo et al. (98.4%) (Guo et al., 2021), and not inferior to conventional fungal culture (100%) and CrAg detection (97.7%) (Yan et al., 2021). Moreover, ROC curve analysis showed that mNGS had a better diagnostic efficiency than conventional culture in the diagnosis of PC (AUC, 0.885 vs. 0.675).

In this study, the detection specimens of mNGS included BALF, commonly used in clinical practice, as well as fresh biopsy lung tissue. Because of the limited number of tissue specimens and the unsatisfactory positive rate of culture, tissue culture is not common (Larkin et al., 2020; Guo et al., 2021) and is mainly used for pathological examination. However, since pathological diagnosis is related to the number of pathogens in the pathological tissue, the quality of specimen staining, and the diagnostic experience of pathologists (Bialek et al., 2002), pathological results may also be false negatives. As observed in Example 1, *Cryptococcus* was not detected in the initial pathological specimen; however, simultaneous LBT mNGS was positive for *C. neoformans*. Re-examination of the deep preparation of histological slices showed a small amount of *Cryptococcus* and the patient was confirmed to have PC, suggesting that mNGS detection could improve the detection rate of fungi in the pathological tissue. In other studies, LBT preserved at -80°C was used for subsequent mNGS detection, and it was found that the diagnostic efficiency of fungi was higher than that of conventional microbial detection (Guo et al., 2021). Since fungal DNA can be detected by mNGS, the fungi identified in formalin-fixed, paraffin-embedded (FFPE) tissues were also confirmed to be highly consistent with the histopathological results, which is of reliable diagnostic value (Larkin et al., 2020; Liu et al., 2024a). In this study, OmNGS and MmNGS were used to detect *Cryptococcus* in fresh lung tissue. The sensitivities for PC diagnosis were 42.9% and 100%, respectively, and the total sensitivity of mNGS was 66.7%. In particular, MmNGS significantly improved the detection ability of *Cryptococcus*. Therefore, a combination of histopathology and mNGS detection may be helpful to further improve the diagnostic rate of fungi (Liu et al., 2024a).

In addition, mNGS has the advantage of identifying *Cryptococcus* as a species, which can better guide precise clinical antifungal regimens. In this study (**Table 2**), except for five cases with false-negative results, 11 cases were identified as *C. neoformans* var. *grubii*, four as *C. neoformans*, and three as *C. neoformans* species complex. Wang et al. (Wang et al., 2022c) reported a case of *C. neoformans* var. *grubii* detected by NGS after the surgical resection of lumbar collateral lesions that significantly improved after fluconazole treatment. Liu et al. (Liu et al., 2024a) reported a case of disseminated cryptococcal infection (prostate and adrenal glands) and suspected involvement of the lungs, brain tissue, and spleen. Pathological examination of the prostate and adrenal tissues revealed fungi, but the fungal species could not be identified. CrAg detection and fungal cultures of the CSF were negative. After the detection of FFPE tissue using mNGS, the patient was identified as *C. neoformans*. Initially, amphotericin B was administered in combination with fluorocytosine. However, an abnormal renal function was observed. Owing to the definite identification of *Cryptococcus*, the drug was changed to high-dose fluconazole, and the patient was finally cured. Therefore, accurate microbiological evidence can assist clinicians in adjusting treatment promptly and improving patient prognosis.

As a highly sensitive method for microbial detection, mNGS provides valuable information for pathogen detection of pathogens (Jin et al., 2022). However, technical difficulties associated with mNGS have limited its clinical application. First, because mNGS requires the extraction of nucleic acids from body samples before pathogen identification, it is difficult to extract DNA from pathogens with thick polysaccharide cell walls, such as *Cryptococcus* and *Aspergillus*. Therefore, a high cell wall-breaking technology is required. Owing to the non-ideal nucleic acid extraction rate, general mNGS technology is not sensitive enough to detect fungi (Peng et al., 2021; Liu et al., 2024b). Wang et al. (2020) found that the detection of serum CrAg in 4 cases of cryptococcal pneumonia was positive, whereas false negative on mNGS occurred in one case. The authors believe that mNGS has no obvious diagnostic advantage for PC. Zhou et al. (Zhou et al., 2021) also reported a case of PC that was negative for *Cryptococcus* in BALF mNGS but positive for serum CrAg. An earlier study showed that the sensitivity of mNGS was only 44.29% in *Cryptococcus* infection cases (Su et al., 2022). These studies indicate that missing the detection of *Cryptococcus* using the general mNGS method may be a problem. Secondly, because the host nucleic acid content in human specimens is high, microbial nucleic acid abundance is relatively low, and the detection sensitivity of pathogens is unstable (Zhou et al., 2021; Shi et al., 2022b; Wang et al., 2022b). Some laboratories use the removal of human nucleic acid technology (reverse pathogen enrichment) when extracting nucleic acids to improve the sensitivity of mNGS (Hasan et al., 2016; Marotz et al., 2018; Gu et al., 2019); however, this method is a double-edged sword, especially if the indiscriminate removal of human nucleic acid is performed, the background microorganisms will be amplified, which can easily cause the loss of low-content pathogen sequences. Certain pathogens, such as *Cryptococcus*, *Aspergillus*, and *Mycobacterium tuberculosis*, may easily be missed. In this study, compared with general mNGS, OmNGS adopted a self-developed homogeneous cell wall-breaking device, which was more effective than the commonly used ultrasonic wall-breaking technology and had controllable quality. Combined with the selective removal of human nucleic acids (Huang et al., 2023), the sensitivity of *Cryptococcus* detection was as high as 61.5%, which was better than those of conventional culture (*P* < 0.05) and general mNGS (Su et al., 2022). To further improve detection sensitivity, MmNGS, which has been further upgraded after technological innovation, has replaced OmNGS. The new MmNGS method can strengthen wall breaking in respiratory specimens, particularly for pathogens (that is, *Cryptococcus, Mycobacterium tuberculosis*) with refractory wall breaking, while considering the stability of RNA pathogens during the extraction process. Compared to the humanized nucleic acid technology adopted by OmNGS, MmNGS adopts forward broad-spectrum enrichment technology to exclude the interference of host nucleic acids (such as biopsy tissue) and background microorganisms (such as sputum and BALF) to avoid detection failure due to the low microbial load in the sample (lower than the detection limit of mNGS). The pathogen detection ability of the low-concentration samples significantly improved. The testing sensitivity of *Cryptococcus* using MmNGS was 100% (10/10), which was significantly (*P* < 0.05) higher than that of OmNGS, while maintaining high specificity (99.0%). Notably, there were five false-negative specimens using OmNGS, including four LBT and one BALF, indicating that tissues were more prone to have missed detection. However, no significant difference in the detection sensitivity between the two specimens (*P* > 0.05) was observed. No missed detections of *Cryptococcus* were observed using MmNGS. However, owing to the limited number of enrolled cases, further studies are required to confirm whether different specimen types have different detection sensitivities.

Previous studies have suggested that *Cryptococcus* occasionally persists in the human oropharynx (Salkowski et al., 1987). Compared with sterile specimens such as blood, CSF, pleural effusion, lung puncture fluid, or biopsy tissue, the positive results of *Cryptococcus* from conventional smears and cultures on sputum and BALF samples often cannot distinguish between contamination, colonization, and infection. Therefore, multiple cultures and channels should be repeated in suspected PC cases. Only multiple positive cultures in non-sterile specimens provided a reference value. However, owing to the low positivity rate of *Cryptococcus* in conventional smears and cultures (Chen et al., 2021), false-positive results in respiratory specimens are rarely found in clinical practice. In our study, three lung puncture fluid samples from three patients with PC and two CSF samples with one blood sample from two patients with disseminated cryptococcal infection tested positive for *Cryptococcus* on culture/smear. Nevertheless, only one BALF sample and one sputum sample from two patients with PC were positive for *Cryptococcus* on culture. Meanwhile, all respiratory specimens from 298 patients without PC were negative for *Cryptococcus* by smear/culture, with a specificity of 100%. For mNGS, such as for CrAg detection (Yan et al., 2021), there are certain false positives; however, the incidence is low. In this study, OmNGS and MmNGS were successively used to detect BALF, and three cases and one case of false positives were found, respectively, with a total false-positive rate of 1.3% (4/298). Su et al. (Su et al., 2022) reported one BALF and two sputum specimens for which *Cryptococcus* read number were detected were considered false positives in three patients with community-acquired pneumonia. Guo et al. (2021) used mNGS on two platforms, Illumina and Nanopore, to identify 133 lung biopsy specimens. During this study, five patients were confirmed to have PC by pathology/culture, and the others were confirmed to have non-PC. Among 128 LBT samples in the non-PC group, there were false-positive results for *Cryptococcus* in two cases (one case of lung cancer and one case of lung shadow) and four cases (two cases of lung cancer, one case of pulmonary tuberculosis, and one case of lung shadow) on the two platforms sequencing respectively. The false-positive rates were 1.5% and 3.0%, respectively, which were slightly higher than that found in our study.

In general, in mNGS, a higher coverage rate, relative abundance, and stringent mapped read numbers are likely to indicate infections (Li et al., 2021). However, the number of reads is affected by the type of specimen, genome size, nucleic acid extraction efficiency, sequencing depth, and platform throughput rate. Therefore, the use of sequence numbers for laboratory-based diagnoses may have led to bias (Li et al., 2020; Zheng et al., 2021). As shown in our study, false positives of *Cryptococcus*, which were detected using mNGS in the BALF of four patients without PC were confirmed by comprehensive clinical judgment. Of these, the sequence numbers (< 10) and relative abundances (< 0.5%) of *Cryptococcus* at the species level were low (**Table 2**). However, the mNGS results of 18 patients with PC who tested positive for *Cryptococcus* showed that the sequence numbers (< 25) and relative abundances (< 0.6%) of *Cryptococcus* at the species level in six cases (33.3%) were also low (**Table 2**). Moreover, the sequence numbers (< 5) were even lower in four cases (**Table 2**), and the results were similar to those of the false positives. In other words, in patients with definite PC, the sequence number and relative abundance of *Cryptococcus* detected by mNGS may also be low, which may be related to refractory wall breakage by *Cryptococcus* and difficulty in nuclide extraction (Li et al., 2020). Thus, the entire clinical picture must be considered when determining the pathogenicity of detected microorganisms such as *Cryptococcus* (Li et al., 2021).

Notably, 75% (3/4) of the cases with false-positive detection of *Cryptococcus* in our study had immunosuppressive diseases/underlying lung diseases, which is similar to the study reported by Guo et al. (2021). In the latter, 50% (1/2) and 75% (3/4) of patients who were false-positive for *Cryptococcus* on the Illumina and Nanopore platforms, respectively, had underlying diseases, including lung tumors and tuberculosis, as observed in our study. Therefore, as shown in Example 2 of our study, even though *Cryptococcus* is detected by mNGS in an immunosuppressed host, the final diagnosis of cryptococcal infection should be determined through a comprehensive clinical data analysis. However, since *Cryptococcus* is an opportunistic infection, the capsule of *Cryptococcus* can resist phagocytosis by macrophages, evade the immune response after the inhalation of *Cryptococcus* propagules into the lungs from the external environment, and establish lung colonization or lymph node complexes, especially in immunosuppressed hosts. This asymptomatic latent lung infection may become active PC or even appear to cause systemic transmission of CM (Montoya et al., 2021). Therefore, for patients detected with *Cryptococcus*, even if the results are considered non-pathogenic fungi at the time, follow-up and close monitoring should be conducted. Serum CrAg levels should be monitored simultaneously, pathological examination should be conducted if necessary, and colonization or pathogenic fungi should be further identified using comprehensive clinical data to avoid missed diagnoses.

This study had some limitations. First, this was a single-center retrospective study with a small number of cases and case selection bias. However, to the best of our knowledge, few studies have specifically evaluated the diagnostic efficiency of mNGS for PC, and the present study enrolled a relatively high number of patients with PC. Secondly, similar to conventional cultures, the results of mNGS cannot distinguish between contaminating, colonizing, and pathogenic fungi, and the final results need to be comprehensively evaluated in combination with clinical data (Su et al., 2022). In this study, four BALF samples proved to be false-positive for *Cryptococcus* and the results were considered non-pathogenic fungi. In one case, as shown in Example 2, the patient had nephrotic syndrome and was initially suspected of *Mycoplasma*-pneumonia with PC but was cured after anti-*Mycoplasma* treatment alone, and the detected *Cryptococcus* was confirmed to be a non-pathogenic bacterium. Third, the testing cost of mNGS is still high (Wang et al., 2022c; Liu et al., 2024a). Most medical units do not have the conditions to carry out this technology, and testing relies on third-party laboratories, which is not conducive to clinical promotion. Routine fungal culture and serum CrAg are still the preferred screening methods in the clinic, and an appropriate pathological biopsy method should be selected according to the clinical requirements and the balance of advantages and disadvantages. When conventional methods cannot meet the needs of diagnosis and treatment of pulmonary diseases, or the disease urgently needs to establish an etiological diagnosis as soon as possible, mNGS can be used as a more rapid auxiliary diagnostic method.

In summary, the diagnostic value of BALF and LBT detection of PC using mNGS was comparable to that of CrAg detection, and the combination of these two techniques can further improve the detection sensitivity. Tissue mNGS combined with pathological examination can increase the positivity rate in patients with negative pathological results. Compared with tissue biopsy, although BALF mNGS is an invasive examination method, it is similar to non-invasive examination, which can avoid lung biopsy damage and is simple and easy to perform. In particular, BALF can be used to detect *Cryptococcus* when early lesions are too small for tissue biopsy or when the primary unit does not have biopsy conditions. In addition, the new MmNGS method used in this study had better diagnostic sensitivity than OmNGS. However, larger prospective studies are required to confirm our findings. Nevertheless, various conventional detection techniques are required for the diagnosis of PC. Clinicians should reasonably choose the detection time of mNGS according to the specific conditions of the patients, especially when the conventional methods cannot be clearly diagnosed. Therefore, mNGS can be used as a better supplementary detection method.

## Data Availability

The original contributions presented in the study are publicly available. This data can be found here: [http://www.ncbi.nlm.nih.gov/bioproject/1175072/ accession number PRJNA1175072].
