# Supplementary material for: Bronchoalveolar lavage fluid and lung biopsy tissue metagenomic next-generation sequencing in the diagnosis of pulmonary cryptococcosis
Source: Front Cell Infect Microbiol. 2024 Oct 29;14:1446814. doi: 10.3389/fcimb.2024.1446814 (PMC11554620; doi:10.3389/fcimb.2024.1446814)
Supplement: Supplementary file 2 [file Table1.docx]

Table S1. Pathogens detected by mNGS in infectious patients without PC

| Pathogen species (n = 166) | n (%) |
| --- | --- |
| Single pathogen  Single bacteria  General bacteria ^a^  *Mycobac**terium tuberculosis* complex  Non-tuberculous *mycobacteria*  *Mycobacterium avium* complex  *Mycobacterium avium*  *Mycobacterium intracellulare*  *Mycobacterium* *abscessus*  Nocardia  *Nocardia mexicana*  *Nocardia abscessus*  *Nocardia cyriacigeorgica*  Atypical pathogen  *Chlamydia psittaci*  *Chlamydia abortus*  *Legionella hackeliae*  *Mycoplasma pneumoniae*  Single fungus  *Pneumocystis jirovecii*  *Aspergillus*  *Aspergillus fumigatus*  *Aspergillus terreus*  *Aspergillus niger*  *Talaromyces marneffei*  *Candida albicans*  Single virus  *Epstein*-*Barr* *virus*  *Human mastadenovirus* B  Mixed pathogens ^b^ | 128 (77.1)  100 (78.1)  43 (43.0)  28 (28.0)  10 (10.0)  6 (60.0)  1 (10.0)  2 (20.0)  1 (10.0)  4 (4.0)  1 (25.0)  1 (25.0)  2 (50.0)  15 (15.0)  12 (80.0)  1 (6.7)  1 (6.7)  1 (6.7)  26 (20.3)  16 (61.5)  6 (23.1)  3 (50.0)  1 (16.7)  2 (33.3)  1 (3.9)  3 (11.5)  2 (1.6)  1 (50.0)  1 (50.0)  38 (22.9) |

Abbreviations: mNGS, metagenomic next-generation sequencing; PC, pulmonary cryptococcosis

^a^ General bacteria included *Pseudomonas aeruginosa* (twelve cases)*, Streptococcus pneumoniae* (five cases)*,* *Staphylococcus aureus* (three cases)*, Haemophilus influenzae* (three cases)*, Moraxella catarrhalis* (one case)*,* *Stenotrophomonas maltophilia* (one case)*,* *Acinetobacter baumannii* (three cases)*,* *Klebsiella pneumoniae* (six cases)*, Streptococcus salivarius* (one case), *Prevotella intermedia* (one case), *Acinetobacter nosocomialis* (one case), *Burkholderia cepacia* complex (one case), *Porphyromonas gingivalis* (one case), *Streptococcus mitis* (one case), *Prevotella buccae* (one case), and *Streptococcus intermedius* (two cases).

^b^ Mixed pathogen (≥two pathogens), including bacteria, fungus, and virus (*Cytomegalovirus* and *Epstein-Barr* virus).
