# Supplementary material for: Bronchoalveolar lavage fluid and lung biopsy tissue metagenomic next-generation sequencing in the diagnosis of pulmonary cryptococcosis
Source: Front Cell Infect Microbiol. 2024 Oct 29;14:1446814. doi: 10.3389/fcimb.2024.1446814 (PMC11554620; doi:10.3389/fcimb.2024.1446814)
Supplement: Supplementary file 3 [file Table2.docx]

Table S2. Mixed pathogens detected by mNGS in infectious patients without PC

| Mixed pathogen (n = 38) | n (%) |
| --- | --- |

| Two types of pathogens  *Acinetobacter baumannii* and *Pseudomonas aeruginosa*  *Acinetobacter baumannii* and *Klebsiella pneumoniae*  *Pneumocystis jerovecii* and *Cytomegalovirus*  *Pneumocystis jerovecii* and *Aspergillus fumigatus*  *Pneumocystis jerovecii* and *Staphylococcus aureus*  *Klebsiella aerogenes* and *Klebsiella pneumoniae*  *Streptococcus constellatus* and *Streptococcus intermedius*  *Streptococcus pseudopneumoniae* and *Streptococcus pneumoniae*  *Mycobacterium avium* and *Mycobacterium abscessus*  *Mycobacterium avium* complex and *Mycobacterium abscessus*  *Burkholderia multivorans* and *Pseudomonas aeruginosa*  *Mycobacterium avium* complex and *Aspergillus flavus/Aspergillus oryzae*  *Legionella pneumophila* and *Klebsiella pneumoniae*  *Enterococcus faecium* and *Escherichia coli*  *Pseudomonas aeruginosa* and *Klebsiella pneumoniae*  *Pseudomonas aeruginosa* and *Aspergillus fumigatus*  *Pneumocystis jerovecii* and *Candida albicans*  *Aspergillus nidulans* and *Candida albicans*  *Pseudomonas aeruginosa* and *Cytomegalovirus*  *Pneumocystis jerovecii* and *Cytomegalovirus*  *Streptococcus oralis* and *Enterococcus faecium*  *Cunninghamella bertholletiae* and *Aspergillus fumigatus*  *Streptococcus oralis* and *Streptococcus mitis*  Three types of pathogens  *Pneumocystis jerovecii*, *Acinetobacter baumannii*, and *Pseudomonas aeruginosa*  *Burkholderia multivorans*, *Enterococcus faecalis*, and *Stenotrophomonas maltophilia*  *Pneumocystis jerovecii*, *Talaromyces marneffei*, and *Aspergillus flavus/Aspergillus oryzae*  *Mycobacterium tuberculosis* complex, *Enterococcus faecalis,* and *Aspergillus fumigatus*  *Enterococcus faecium, Mycobacterium avium* complex, and *Aspergillus flavus/Aspergillus oryzae*  *Pneumocystis jerovecii, Cytomegalovirus,* and *Epstein-Barr virus*  Four types of pathogens  *Acinetobacter bereziniae*, *Acinetobacter nosocomialis*, *Pseudomonas aeruginosa*, and *Aspergillus terreus*  More than four types of pathogens (micropopulation *) | 27 (71.1)  2  1  3  2  1  1  1  1  1  1  1  1  1  1  1  1  1  1  1  1  1  1  1  6 (15.8)  1  1  1  1  1  1  1 (2.6)  1  4 (10.5) |
| --- | --- |

Abbreviations: mNGS, metagenomic next-generation sequencing; PC, pulmonary cryptococcosis

*Micropopulation means mixed infection with multiple anerobic bacteria.
